# An Improved Density Peak Clustering Algorithm for Multi-Density Data

**DOI:** 10.3390/s22228814

**Published:** 2022-11-15

**Authors:** Lifeng Yin, Yingfeng Wang, Huayue Chen, Wu Deng

**Affiliations:** 1 School of Software, Dalian Jiaotong University, Dalian 116028, China; 2 School of Computer Science, China West Normal University, Nanchong 637002, China; 3 School of Electronic Information and Automation, Civil Aviation University of China, Tianjin 300300, China; 4 Traction Power State Key Laboratory, Southwest Jiaotong University, Chengdu 610031, China; 5 Guangxi Key Laboratory of Hybrid Computation and IC Design Analysis, Guangxi University for Nationalities, Nanning 530006, China

**Keywords:** density peak, cluster, local density

## Abstract

Density peak clustering is the latest classic density-based clustering algorithm, which can directly find the cluster center without iteration. The algorithm needs to determine a unique parameter, so the selection of parameters is particularly important. However, for multi-density data, when one parameter cannot satisfy all data, clustering often cannot achieve good results. Moreover, the subjective selection of cluster centers through decision diagrams is often not very convincing, and there are also certain errors. In view of the above problems, in order to achieve better clustering of multi-density data, this paper improves the density peak clustering algorithm. Aiming at the selection of parameter *d_c_*, the K-nearest neighbor idea is used to sort the neighbor distance of each data, draw a line graph of the K-nearest neighbor distance, and find the global bifurcation point to divide the data with different densities. Aiming at the selection of cluster centers, the local density and distance of each data point in each data division is found, a *γ* map is drawn, the average value of the *γ* height difference is calculated, and through two screenings the largest discontinuity point is found to automatically determine the cluster center and the number of cluster centers. The divided datasets are clustered by the DPC algorithm, and then the clustering results are perfected and integrated by using the cluster fusion rules. Finally, a variety of experiments are designed from various perspectives on various artificial simulated datasets and UCI real datasets, which demonstrate the superiority of the F-DPC algorithm in terms of clustering effect, clustering quality, and number of samples.

## 1. Introduction

Cluster analysis [1] is a method of classifying similar samples of a dataset into several classes. The set of clusters produced by clustering analysis is called a cluster, and in this context, different clustering methods may produce different clusters on the same dataset. The division is not performed by people, but by a clustering algorithm. Therefore, how to measure the similarity between samples is the key problem of a clustering algorithm. Clustering [2], as an unsupervised learning process, has also been applied in various fields [3,4,5,6], such as image recognition, document search, intrusion detection, and sentiment analysis. At present, the most classic and commonly used clustering algorithms [7,8] include density-based clustering algorithms, hierarchical clustering algorithms, grid-based clustering algorithms, graph-theory-based clustering algorithms, and some other optimization algorithms [9,10,11,12,13,14,15].

The hierarchical clustering algorithm, also known as the tree clustering algorithm, has the advantage of being able to clearly express the hierarchical relationship between clusters. The grid-based clustering method is suitable for any attribute dataset, and the algorithm running time depends on the grid cell size, which can greatly improve the computational efficiency. The clustering method based on graph theory can transform the clustering problem into a graph partitioning problem, which is more suitable for discovering clusters with irregular shapes in the dataset. The density-based clustering algorithm clusters according to the distance between objects and replaces the similarity of data with density; the advantage is that it can filter noise or outliers and can find clusters of any shape.

The density peak clustering algorithm [16] (denoted as DPC) is an emerging density-based clustering algorithm, proposed by Rodriguez et al. in 2014, and the algorithm calculates the local density of data points and the distance of data points by setting the value of the parameter truncation distance *d_c_*, and then draws a decision diagram to observe and determine the cluster center and the number of cluster centers. However, this clustering method has certain defects. First, the subjective selection of cluster centers by the naked eye will lead to inaccurate selection of cluster centers, which will lead to weaker robustness of the clustering algorithm. Secondly, the parameter truncation distance *d_c_* only selects a unique value, which is neither objective nor scientific for multi-density datasets, and the selection of *d_c_* also directly affects the final result of clustering. The selection of *d_c_* depends on the distance between samples. When the distance difference within each cluster is obvious, the selection of the neighborhood truncation distance *d_c_* is seriously affected. In this case, the DPC algorithm cannot obtain a good clustering effect. Aiming at the problem of the selection of the parameter *d_c_* of the DPC algorithm, reference [17] optimized the parameter *d_c_* by using the maximum difference method of adjacent elements. However, under the condition of multi-density data, the parameters optimized according to the local distance of data points cannot obtain stable clustering effect. Reference [18] combined the DPC algorithm with K-means to optimize the initial clustering center point, so as to better achieve the local optimal clustering effect and greatly reduce the number of clustering iterations. However, under the condition of multi-density data, the data center of gravity will be affected and the clustering results will be affected. Reference [19] proposed a DPC algorithm based on weighted K-nearest neighbors and geodesic distance (DPC-WKNN-GD), using the idea of weighting to improve the parameter *d_c_* to improve the clustering performance of manifold and non-manifold datasets. Reference [20] also proposed an adaptive density peak algorithm combined with the whale optimization algorithm to obtain the best cut-off distance *d_c_*, which also strengthened the accuracy of the original parameter calculation. These two algorithms also optimize the clustering parameters so that the uniqueness of the parameters cannot be applied to multi-density datasets. For datasets with multi-density, reference [21] proposed a heuristic filter based on density peak clustering (ADPCHFO) for adaptive weighted oversampling of unbalanced datasets, which can solve both the inter-class and intra-class imbalances question. Reference [22] proposed an improved hierarchical clustering algorithm to solve data with multi-density, first using fuzzy pre-clustering division and then using the Jaccard similarity coefficient for fusion. Reference [23] uses the idea of region division grid to solve the multi-density problem. However, the above methods need to use many parameters or the computational complexity is large, so that the accuracy of the clustering model will be affected by multiple parameters. When encountering a large number of datasets, the accuracy of the clustering model will be affected by multiple parameters. Faced with the high computational complexity of density peak clustering in large datasets, reference [24] proposed a density peak clustering algorithm based on sparse search and K-d tree, the algorithm proposes a sparse search strategy to speed up the calculation of relative separation and greatly reduce the time complexity of the algorithm; Faced with the problem that it is difficult to accurately find the cluster centers in large datasets, reference [25] proposed a method based on the Gini coefficient and K-nearest neighbors to calculate the cut-off distance to automatically determine the cluster centers. Reference [26] automatically determined the number of inflection points in the decision diagram according to the characteristics of different datasets and can further determine the number of cluster centers without manual intervention. Reference [27] uses a continuous function to distinguish different data point densities to automatically determine the clustering center. The above four methods solve the problem of cluster center selection and time consumption of the DPC algorithm for large datasets. However, in the face of a large number of multi-density data, the above methods are difficult to accurately select the cluster centers, and there are few domestic and foreign scholars on this aspect. In the face of high-dimensional complex data, references [28,29] use the method of calculating the feedback value combined with the support vector machine and the method of calculating the order similarity between samples to process the high-dimensional data, respectively. However, for complex data with multiple densities, a large number of calculations are also a test of time consumption. In addition, some other methods were also proposed in recent years [30,31,32,33,34,35,36,37,38]

For the above problems and defects, this paper improves the DPC algorithm for multi-density data and records it as F-DPC. The algorithm solves the uniqueness and sensitivity of the original algorithm parameters under the condition of multi-density data, and also solves the problem of poor clustering effect caused by subjective selection of cluster centers. The main work is as follows:

(1) In order to solve the problem of unsatisfactory clustering effect caused by the uniqueness of data parameters of multi-density, the distance matrix is obtained by the distance between any two points of each data point, and the K-nearest neighbor matrix is obtained by row in ascending order. Draw a line graph of the K-nearest neighbor distance according to the parameter *k*, find the global bifurcation point for division, and obtain *D* = {*D*1, *D*2, ..., *Dm*}, where m is the number of divisions. Calculate the corresponding parameter truncation distance *d_ci_* for each *d_ci_*, where *i* ∈ [1,*m*].
(2) In order to solve the problem of subjective selection of cluster centers, for each *D_i_*, calculate the local density *ρ_j_* and data point distance *δ_j_* of each data point, calculate the product of the two *γ_j_*_,_ and sort them in ascending order. Finally, draw the scatter plot of each data *γ_j_*, calculate the height difference between two adjacent points, calculate the average height difference, and select the point higher than the average height difference as the center point of the preliminary cluster. Screen again according to the preparatory cluster center points to determine the cluster center and the number of clusters of each *D_i_*.
(3) Each *D_i_* performs the DPC algorithm according to the obtained cluster center and *d_ci_* to obtain a new cluster. Finally, the clusters are merged through the fusion rule to obtain the final cluster.
(4) Comparative experiments are carried out from various perspectives on various artificial simulated datasets and UCI real datasets. From the perspective of various measurement indicators of clustering, the clustering quality of the F-DPC algorithm is the best; however, from the perspective of time consumption, the time consumption of the F-DPC algorithm increases with the increase in the amount of data, but the increase level is in the middle.

The rest of this paper is organized as follows: Section 2 briefly introduces the relevant knowledge of the density peak clustering algorithm; Section 3 introduces the idea of the F-DPC algorithm, the selection of parameters, the selection of cluster centers, and the corresponding pseudocode and time complexity; Section 4 introduces the clustering performance of each algorithm on various numbers of artificially simulated datasets and UCI real datasets, and conducts experimental analysis from multiple perspectives; finally, Section 5 contains the conclusions of the work.

## 2. DPC Algorithm

The DPC algorithm [39] considers local density and relative distance to draw a decision graph, quickly identify cluster centers, and complete clustering. This section mainly introduces the idea of the DPC algorithm [40], the formula for local density calculation [41], the selection of cluster centers [42], the selection of DPC algorithm parameters *d_c_*, and the DPC algorithm process.

### 2.1. DPC Algorithm Idea

The basic idea of the DPC algorithm is to find high-density areas separated by low-density areas and draw a decision diagram by calculating the local density of all data points and the distances of all data points, finding the cluster center, and then performing clustering according to the truncation distance parameter *d_c_*.

### 2.2. DPC Algorithm Formula

The main work of the DPC algorithm is to calculate the local density, which can be calculated by Formula (1).

(1) $$\rho_{i}=\sum_{j\neq i}\chi (d_{ij}-d_{c})$$

$$\chi (x)=\left\{\begin{array}{l} 1,\;if\;x<0\\ 0,\;if\;x\geq 0 \end{array}\right.$$

Among the equation components, *d_ij_* represents the Euclidean distance between data point *i* and data point *j*; the parameter *d_c_* represents the cut-off distance (the calculation method of *d_c_* is given in subsection C); and *ρ_i_* is the local density of data point *I*; that is, draw a circle with data point *i* as the center and *d_c_* as the radius, and the number of objects whose Euclidean distance between data point *i* and the rest of the objects is less than the cut-off distance radius *d_c_.*

The distance *δ_i_* of each data point *i* is the cut-off distance between the data point whose density is greater than that of *I*, and the cut-off distance from *i* is the smallest and is calculated according to Formula (2).

(2) $$\delta_{i}=\underset{j:\rho_{j}>\rho_{i}}{\min}(d_{ij})$$

If the local density of the current data point i is the largest, then the distance calculation formula is calculated using Formula (3).

(3) $$\delta_{i}=\underset{j}{\max}(d_{ij})$$

### 2.3. Selection of Cluster Centers

**Figure 1:**
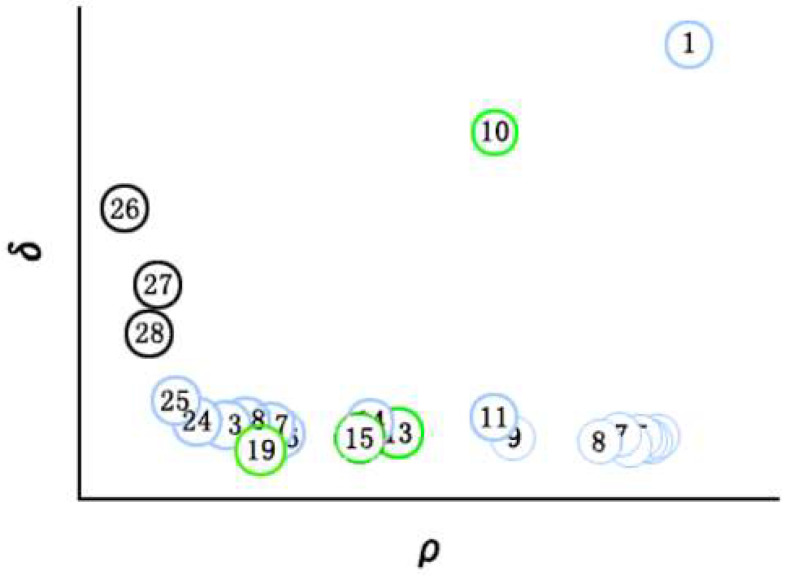
Decision Diagram Example.

Calculate the *ρ_i_* and *δ_j_* of each data point and draw a scatter plot with *ρ_i_* as the abscissa and *δ_i_* as the ordinate, which are collectively referred to as the decision map. Only when the local density *ρ* of the data points and the distance *δ* of the data points are relatively large, it is suitable as the cluster center point; lower *ρ* and higher *δ* act as noise points or outliers. After finding the cluster center point, the remaining points are assigned to the cluster where the data point closest to the current point and with a density greater than it is located. In the decision diagram example shown in Figure 1, data point 1 and data point 10 are suitable as cluster center points, and the remaining points are assigned clusters according to the above principles, the circles with numbers in the same color represent the same class, and the numbers in the black circles represent outliers.

### 2.4. Selection of DPC Algorithm Parameters

The DPC algorithm [16] has only one truncation distance parameter *d_c_*, and the selection size is particularly important. If the setting is too large, it is easy to divide the original multiple clusters into one cluster. If the setting is too small, it is possible to divide the classes that should be in one cluster into two categories. The DPC algorithm summarizes experience and concludes that the value of *d_c_* is the best value when the number of data points with an average surrounding distance of each data point less than *d_c_* accounts for 1% to 2% of the total number of data points.

### 2.5. Steps of DPC Algorithm

The steps of the DPC algorithm are as follows:

(1) Calculate the distance between any two points.
(2) Estimate the global parameter *d_c_* value.
(3) Calculate the local density *ρ_i_* of each point.
(4) Calculate the data point distance *δ_i_* for each point.
(5) Draw a decision diagram according to (3) and (4).
(6) Estimate the cluster center and the number of clusters.
(7) The remaining points are assigned to the cluster of data points that are closest to the current point and whose local density is greater than that.

## 3. F-DPC Algorithm

For the multi-density data, the DPC algorithm parameter *d_c_* is unique, and the cluster center selection is subjective. This section improves the DPC algorithm and proposes the F-DPC algorithm.

### 3.1. The Basic Idea of F-DPC Algorithm

This section describes the specific operation of the F-DPC algorithm.

First, the F-DPC algorithm uses the idea of the K-nearest neighbor algorithm [43] to obtain the Euclidean distance of the *k*-th nearest neighbor data of each data point, arranges the data points in ascending order according to the obtained distance from small to large, and draws a distance line graph. The data points with the same density tend to be flat in the distance line chart, while the data with different densities will obviously have a bifurcation point in the graph. Find the data bifurcation points in the graph to divide the data of different densities. This method avoids the problem of affecting the clustering effect because there is only one parameter globally under the condition of multi-density data.

Secondly, the truncation distance parameter *d_c_* of the F-DPC algorithm is obtained by calculation, and its calculation rule is the following: First, count the number of points whose surrounding distance is less than *d_c_*, then sum and average, and satisfy that the average accounts for 2% of the sum of all points. The parameter *d_c_* value at this time is the cut-off distance we need. The *d_c_* calculated by this method gets rid of the defect of manually setting the parameters empirically.

Again, calculate the local density *ρ_i_* of each data point and the data point distance *δ_i_* (i ∈ [1,*n*], *n* represents the number of data points); after normalizing *ρ_i_* and *δ_i_*, take the product to obtain γ_i_, arrange all γ_i_ in ascending order, and then draw a γ scatter plot, calculate the height difference in adjacent data points in the γ scatter plot and the average value of the height difference $\bar{hv}$, and select the data points higher than $\bar{hv}$ as the pre-selected cluster center. Calculate the height difference between adjacent points from the preselected cluster centers and find the point with the largest height difference, that is, the largest discontinuity point, so as to determine the cluster center and the number.

Finally, according to the obtained cluster centers and cut-off distances, the divided data are clustered, respectively, the clustering results are fused according to the cluster fusion rules, and the obtained result is the final cluster.

**Figure 2:**
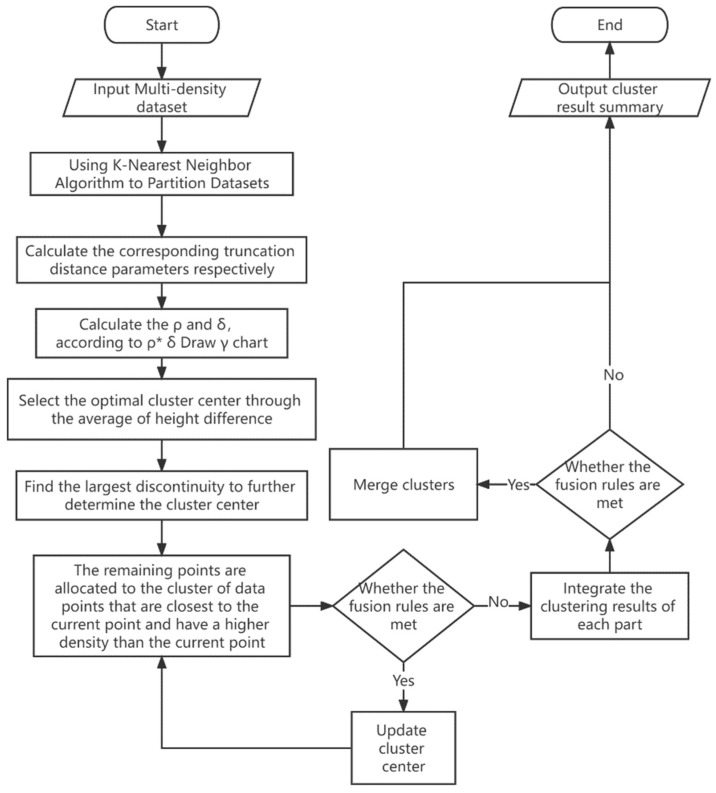
The flow of F-DPC algorithm.

The flow chart of the F-DPC algorithm is shown in Figure 2.

### 3.2. F-DPC Algorithm Design

#### 3.2.1. K-Nearest Neighbor Algorithm to Divide Dataset

This section proposes the idea of using the K-Nearest Neighbors algorithm to divide the known dataset in preparation for clustering. First, the Euclidean distance between each data point and the rest of the points is calculated, and a distance matrix is obtained. Set a parameter *k*, record the Euclidean distance from each point to the *k*-th nearest neighbor data point, arrange them in ascending order, and draw a line graph of the K-nearest neighbor distance to divide the dataset.

Step 1: Calculate the Euclidean distance from each point in the dataset to the rest of the points, where *n* represents the number of datasets, *dist*(*i*,*j*) represents the Euclidean distance from the *i*-th data to the *j*-th data, and the K-nearest neighbor distance matrix is obtained according to Formula (4), which is a real symmetric matrix.

(4) $$DIST_{n\times n}=\{dist(i,j)|1\leq i\leq n,1\leq j\leq n\}$$

Step 2: Sort each row of data in the distance matrix $DIST_{n\times n}$ in ascending order to obtain a new matrix $KDIST_{n\times n}$. As shown in Formula (5), through this matrix, it is convenient to find the *k*-th nearest neighbor distance from each data point to the rest of the points at the same time, and it is convenient to draw a K-nearest neighbor line graph.

(5) $$KDIST_{n\times n}=\{kdist(i,k)|1\leq i\leq n,1\leq k\leq n\}$$

Step 3: For the sorted matrix, select the *k*-th nearest neighbor distance of each point at the same time, where the *k* value is generally selected within 10% of the total number of datasets.

Step 4: Arrange the *k*-th nearest neighbor distance of each data point in ascending order, and then obtain the new index number of the data point at the same time. Taking the new index number of the data point as the abscissa value, and the *k*-th column value in $KDIST_{n\times n}$ corresponding to this data point as the ordinate value, draw a K-distance line graph.

Step 5: Find the bifurcation points (that is, the points with obvious height difference), bind the left and right parts of each bifurcation point by index and divide the dataset, and plan the data with the same density together to complete the dataset division.

The pseudocode related to the Algorithm 1 is as follows.
**Algorithm 1** Divide_Datasets.
Input: Multi-density Dataset *D*Output: Dataset summary after dividing the dataset *DD*1 *X* = read(*D*)  // read data into *X*2 *disMat* = squareform(*X*)  // Calculate the distance between any two points.3 for *I* in *disMat*4    *i*.sort()  // Sort each row of data5    *array*.append(*i*[*k*])  // The kth nearest neighbor distance of each point is stored in an *array.*6 Use *plt*.plot to draw a distance line chart on the *array*;7 Calculate *array*[*i* + 1]-*array*[*i*] successively from the figure to find the point with obvious height difference, and mark array[*i* + 1] as the bifurcation point;8 Use index binding to divide the dataset into two parts left and right of the bifurcation point;9return *DD*

#### 3.2.2. Selection of Parameter Cut-Off Distance *d_c_*

The F-DPC algorithm borrows the method of selecting the parameter *d_c_* from the DPC algorithm (mentioned in the previous Section 2.4), and then calculates the superior *d_c_* from the divided datasets with different densities. The specific parameter calculation process is as follows.

Assuming a total of *n* data, first calculate the Euclidean distance between any two points of the data point to obtain a real symmetric distance matrix, and count the total amount of data distance (note: the distance of the data point itself is not the total distance), that is, *n* × (*n* − 1); after the total distance is obtained, calculate 2% of the total distance position *p*, that is, *p* = *n* × (*n* − 1) × 0.02, arrange the distance matrix in ascending order and turn it into an ascending table *t* of length 1 × *n*^2^, and according to the previous position *p*, find the data point whose number of corresponding points accounting for 2% of the number of all points is *d_c_*, *d_c_* = *t* [*p* + *n*] (in the ascending distance list, the distance of the first *n* items is the distance of the data point itself, so *n* needs to be added).

The pseudocode related to the Algorithm 2 is as follows.
**Algorithm 2** Parameter_Selection.
Input: Currently partitioned dataset *dd*Output: Parameter *dc*1 *disMat* = squareform(*dd*)  // Statistical data distance total to get distance matrix2 *position* = int(*n* * (*n − 1*) * *per*/100)  //*per = 2%*, N represents the number of currently divided datasets, and records the selected truncation distance *dc* position3 *dc* = sort(*t*)[*position + N*] 4 return *dc  / /* return parameter

#### 3.2.3. The Selection of Cluster Centers and the Number of Centers

This paper proposes to use *γ_i_*, that is, the product of *ρ_i_* and *δ_j_*, to comprehensively consider the cluster centers [44], and use Formula (6) to calculate. Normalization [45,46] is the process of adjusting measurements in different scales to the same scale and can be even more complex to make the probability distribution of the adjusted values consistent. In order to eliminate the dimensional influence between the feature data, it is necessary to normalize the features *ρ_i_* and *δ_j_*. This paper adopts the Min-Max Normalization method shown in Formula (7) to solve the comparability between feature indicators, where *x* represents the current data, min represents the smallest value in the current data, max represents the largest value in the current data, and x* represents the normalized data value size. After the original data are normalized, each index is in the same order of magnitude for comprehensive comparative evaluation.

(6) $$\gamma_{i}=\rho_{i}\delta_{i}$$

(7) $$x^{*}=\frac{x-\min}{\max-\min}$$

Taking the data index number corresponding to the result of the ascending order of the *γ_i_* data as the abscissa, and the *γ_i_* data as the ordinate, draw the corresponding *γ* scatter diagram. The first step is to calculate the height difference $hv_{i+1}$ before and after *γ_i_*_+1_ and *γ_i_* according to Formula (8), calculate the average height difference $\bar{hv}$ according to Formula (9), and filter out the points greater than the average height difference to obtain a new set {r_1_,r_2_,...r_m−1_,r_m_}, where m is the number of data points greater than the average height difference, the data in the set is sorted in ascending order, and this set is used as the preliminary clustering center. The second step is to determine the cluster center according to the preparatory cluster center. The dataset after the default division of this algorithm is divided into at least two categories. Therefore, r_m_ must be the cluster center, and the remaining cluster centers are selected from the set {r_1_,r_2_,...r_m−1_}. Continue to calculate the height difference between two adjacent points in this set. If the object *i* + 1 is selected, the maximum height difference is obtained at this time, that is, *γ_i_*_+1_ − *γ_i_* = max (hv). Then, the cluster center selects all points after *γ_i_*_+1_ in the set {r_1_,r_2_,...r_m−1_} as the cluster center. Through the screening of the two cluster centers, the cluster centers can be selected more advantageously.

(8) $$hv_{i+1}=\gamma_{i+1}-\gamma_{i}(i=0,1,\ldots ,n-2)$$

(9) $$\overset{-}{hv}=avg(\sum_{i=1}^{n-1}hv_{i})$$

The pseudocode related to the Algorithm 3 is as follows.
**Algorithm 3** Select_Cluster_Center.
Input: Currently partitioned dataset *dd*Output: *cc*1 Statistical local density is sorted and calculated and stored in *normal_den*;2 for *i* in *dd*
3    Statistical local density is sorted and calculated and stored in *normal_dis*;4    *gama* = normal_den*normal_dis  // Preparing the product of the two parts for drawing the γ graph;5    Use *plt*.plot to draw a gama graph for γ;6      for j in range(len(gama))7           *R*.append(*gama*[*i*]-*gama*[*i + 1*])  //The height difference between front and rear is stored in *R*.8           Calculate height difference mean in *R*, filter out the pre-cluster center is stored in *K*;9       Compare the height difference in *K*, find the maximum height difference, and select the larger data point as the maximum discontinuity point;10       Screen out the points greater than or equal to the maximum discontinuity point as the final cluster center and store it in *cc*, and update the labels of the dataset *dd*;11   end for
12 end for 
13 return *cc*

#### 3.2.4. Cluster Fusion

Clustering each partition of the dataset, the clustering results appear as follows. Too many clusters on the divided dataset will generate redundant clusters; when selecting cluster centers, the algorithm defaults to at least two cluster centers, but it cannot be ruled out that the divided dataset has only one class or that there are more predicted classes than the original dataset. In view of the above clustering results, this paper uses the idea of boundary sample optimization [47] to fuse the two types of clusters. The fusion of clusters needs to meet two conditions, first to determine whether the category is an adjacent cluster, and then to determine whether the fusion rules are satisfied. The judgment conditions of adjacent clusters are the following: assuming that *p* and *q* are data points, *C*1 and *C*2 are clusters, and satisfy $\exists p\in C1,q\in C2,dis\left(p,q\right)\ll d_{c}$ holds, then *C*1 and *C*2 belong to adjacent clusters, and *p* and *q* belong to adjacent samples. The judgment condition of the fusion rule is that the proportion of the number of statistical adjacent samples to the total number of samples in the two adjacent clusters is more than 2%.

After completing the clustering of each division of the dataset, for the clustering results of each division, the two clusters that satisfy the fusion rules are fused. The cluster center of the fused cluster is the cluster center with a larger *γ* value, and then re-clustering is performed until there are no clusters that meet the fusion rules, and the fusion process of this dataset division ends. Integrate the fused results of each division of the dataset, and then perform the fusion rule detection again. If the fusion rules are met, merge the two clusters until they cannot be fused. The final result is the clustering result of the entire dataset.

The pseudocode related to the Algorithm 4 is as follows.
**Algorithm 4** Cluster_Fusion.
Input: Dataset *pp* that needs fusion detectionOutput: Fusion clustering results1 for *I* in *pp //* Count any two clusters in the current dataset.2    for *j* in *pp*3    if there are two points with distance < *d_c_* and from two different labels4         marked as adjacent samples and adjacent clusters;5   end if6 end for7 if meet the fusion rules(The number of adjacent samples accounts for more than 2% of the total number of two adjacent clusters)8   Update the cluster center cc to re-cluster, and perform fusion detection again after re-labeling;9   Update dataset labels;10 end if11 return *cluster  //* Return clustering results.

### 3.3. Time Complexity Analysis of F-DPC Algorithm

The F-DPC algorithm includes five parts, dataset division, parameter selection, cluster center selection, DPC rule clustering, and cluster fusion, where *m* represents the number of data points in the overall dataset, *n* represents the number of data points that are currently divided into the dataset, *k* represents the number of divided datasets, and *r* represents the number of preselected cluster centers: *m* >= *n*, *n* >= *k*, *n* >= *r*.

(1) Dataset division needs to traverse all data, count the distances of all data points to obtain a distance matrix and sort, where *m* is the total amount of data, so the time complexity is O(*m*^2^).
(2) In the selection of parameters, the parameters are obtained by traversing the data distance of the current dataset, where *n* is the number of the current dataset, so the time complexity is O(*n*^2^).
(3) In the selection of cluster centers, it is necessary to traverse all data points of the current divided dataset to calculate the *γ* value, and then traverse and draw the *γ* graph again to filter the cluster centers. The time complexity is O(*n*^2^), and then the final cluster centers are selected from the preselected cluster centers, where *r* represents the number of preselected cluster centers, *r* <= *n*, so the algorithm time complexity of the whole process is O(*n*^2^ * *r*).
(4) DPC rule clustering needs to traverse the local density and data point distance of each data point. The traversal length is also the total number of data points in the current dataset *n*, and the algorithm time complexity is O(*n*).
(5) In the process of cluster fusion, the process of finding adjacent clusters, traversing the distance of any current dataset data points to judge adjacent clusters and adjacent samples, the algorithm time complexity is O(*n^2^*).

This algorithm is executed sequentially. For the F-DPC algorithm, (3)–(5) also need to perform the same operation on each divided dataset, and the time complexity should be multiplied by the number of divided datasets *k*, where *r* and *k* of this algorithm are at least 2 by default, and the time complexity of this algorithm is O(*n*^2^ * *r* * *k*).

### 3.4. Algorithm Example Analysis

**Figure 3:**
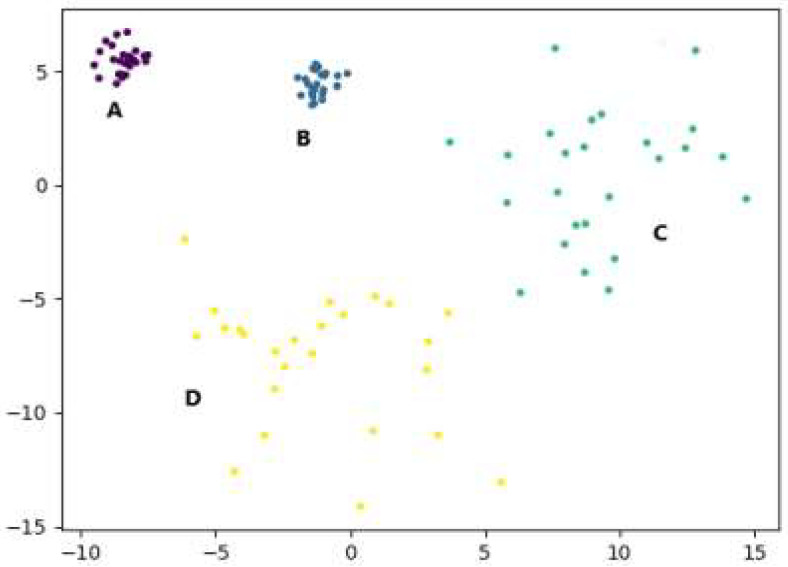
Scatter plots of artificially simulated datasets.

This section uses the artificial simulation dataset to illustrate the working process of the F-DPC algorithm. The dataset data have a total of 100 two-dimensional data, four categories, and each category accounts for 25 samples. Among them, the density of class A and class B are close, the intra-group distance is small, the density of class C and D is close, and the intra-group distance is large. The density of this artificial simulation dataset is not uniform, and the corresponding scatter plot is shown in Figure 3.

**Figure 4:**
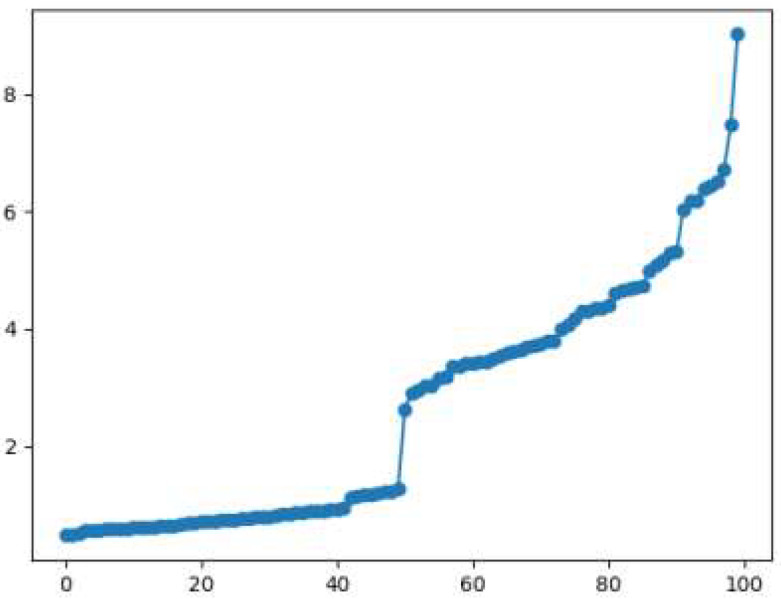
K-Nearest Neighbor Line Chart (*n* = 100).

Taking the dataset in Figure 3 as an example, the K-distance line chart obtained from steps 1 to 4 in the first subsection of Section B is shown in Figure 4. The abscissa in the figure is the index number of the sorted data points, and the ordinate is the 10th nearest neighbor distance. It can be clearly seen from the figure that the sorted data points have an obvious bifurcation point at position 50. This bifurcation point is the transition from the data of one density to the data of another density, with the boundary of number 50, dividing the dataset into two parts: data1 and data2.

**Figure 5:**
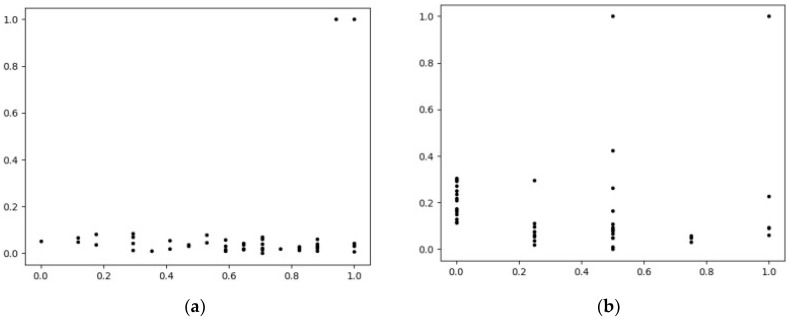
Decision diagram (*n* = 50). (a) decision diagram of data1; (b) decision diagram of data2.

Calculate the local density *ρ_i_* and data point distance *δ_j_* of each data point, and then draw the decision diagrams of data1 and data2 with *ρ_i_* as the abscissa and *δ_j_* as the ordinate, respectively, as shown in Figure 5a,b.

**Figure 6:**
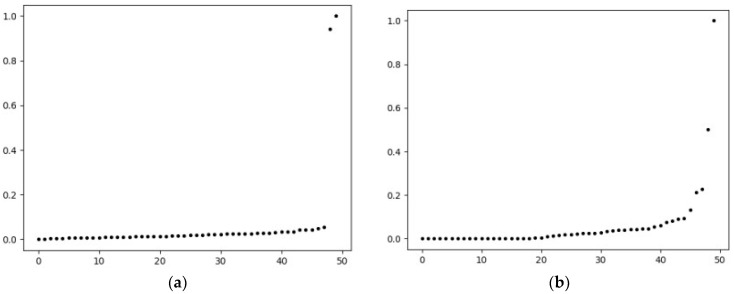
*γ* scatter plot (*n* = 50). (a) *γ* scatter plot of data1; (b) *γ* scatter plot of data2.

There is a certain error in the cluster center points seen by the naked eye in the figure. According to the method of selecting the parameter *d_c_* in this paper, F-DPC calculates the product of *ρ_i_* and *δ_j_* and draws the scatter plots of the *γ* values of data1 and data2 in ascending order, as shown in Figure 6a,b.

**Figure 7:**
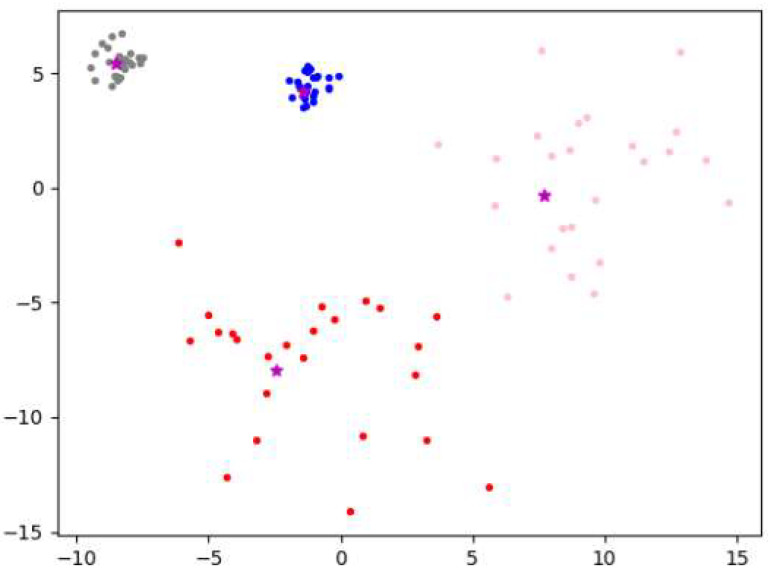
The *γ* scatter plot of data1 and data2 (*n* = 50).

The F-DPC algorithm uses the DPC algorithm to cluster data1 and data2 according to the found cluster centers and numbers, and then fuses them according to the fusion rules. The final clustering result of the F-DPC algorithm is shown in Figure 7. It can be seen from the figure that the initial cluster center is basically in the center of each cluster, and the clustering effect is very good.

This example analysis shows that F-DPC has a good clustering effect.

## 4. Discussion

This section introduces the evaluation indicators to measure the quality of the clustering algorithm, conducts experiments on artificial simulated datasets and UCI real datasets, designs a variety of experimental methods, and analyzes the experimental results from multiple perspectives to illustrate the superiority of the F-DPC algorithm.

### 4.1. Algorithm Evaluation Metrics

The evaluation index of the algorithm [48,49,50,51,52] selects the precision rate (precision), the recall rate (recall), the accuracy rate (ACC), the harmonic mean (F1) of the precision rate and the recall rate, the adjusted Rand coefficient (ARI), the adjusted mutual information (AMI), Fowlkes–Mallows Index (FMI), and Normalized Mutual Information (NMI). What they represent is as follows.

Precision refers to the proportion of the samples that are actually positive among all the samples that are judged to be positive and reflects the error rate of the prediction results. The calculation is shown in Formula (10).

(10) $$P=\mathrm{TP}/\left(\mathrm{TP}\;+\;\mathrm{FP}\right)$$

Recall refers to the proportion of positive samples among all the actual positive samples, which reflects the missed detection rate of the prediction results. The calculation formula is shown in (11).

(11) $$R=\mathrm{TP}/\left(\mathrm{TP}\;+\;\mathrm{FN}\right)$$

Accuracy refers to the proportion of all samples that are correctly classified. The calculation formula is shown in (12).

(12) $$\mathrm{ACC}=\left(\mathrm{TN}+\mathrm{TP}\right)/\left(\mathrm{TP}+\mathrm{FP}+\mathrm{TN}+\mathrm{FN}\right)$$

F1 is the harmonic mean of precision and recall, and the calculation formula is shown in (13). Its value is between 0 and 1, and the closer to 1, the better the clustering effect.

(13) $$F1=\left(2\times P\times R\right)/\left(P+R\right)$$

Among the equation components, TP is the number of true positives, FP is the number of false positives, TN is the number of true negatives, and FN is the number of false negatives.

ARI is used to measure the degree of agreement between two distributions. The value range is [−1,1]. The closer the value is to 1, the better.

AMI is used to measure the degree of agreement between two distributions. The value range is [−1,1]. The larger the value, the more consistent the clustering effect is with the real situation.

FMI is the result of the geometric mean of the recall rate and precision rate calculated from the clustering result and the real value. The value range is [0,1], and the closer to 1, the better.

NMI is used to measure the similarity of two clustering results. The value range is [0,1]. The higher the value, the more accurate the division.

### 4.2. Analysis of Experimental Results on Artificial Synthetic Datasets

In this section, experiments are carried out on the F-DPC algorithm, the clustering algorithm DPC [17] with improved parameter *d_c_*, the algorithm combining DPC and K-means [18], the DPC algorithm and the K-means algorithm.

#### 4.2.1. Experimental Analysis from the Perspective of Clustering Effect

**Figure 8:**
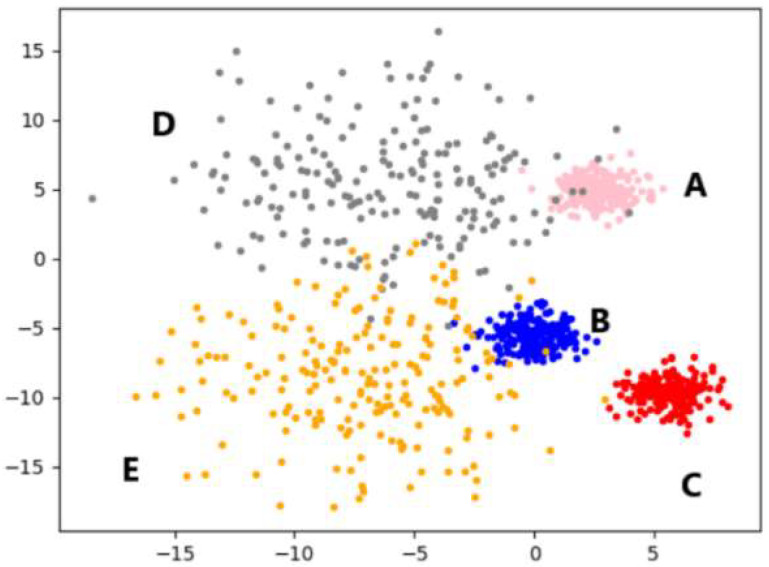
Multi-density dataset (*n* = 1000, C = 5).

The experiments in this section use the multi-density dataset shown in Figure 8, the data size is 1000, the feature is 2, and the category is 5. Experiments were carried out on five algorithms, the clustering results were visualized, and letters were marked on each category position. The position markers of all visualization graphs are consistent with the position markers in Figure 8, and each algorithm is analyzed from the perspective of clustering effect.

**Figure 9:**
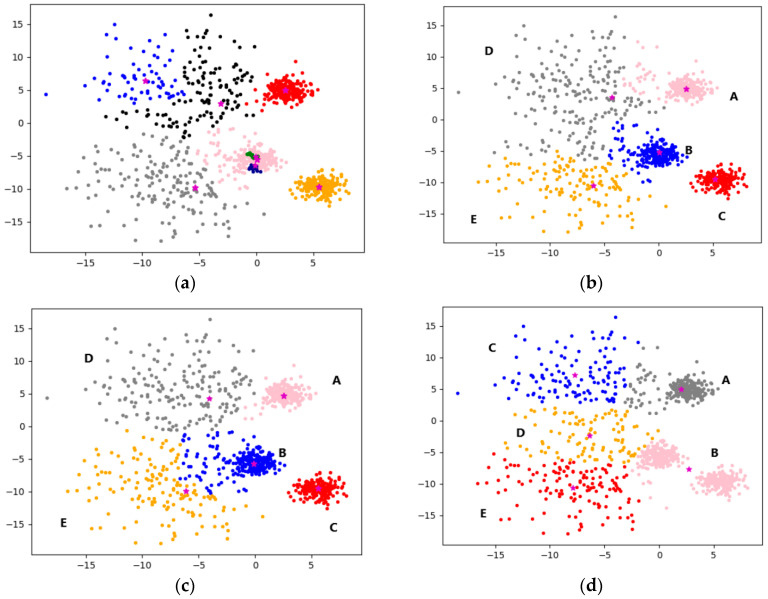

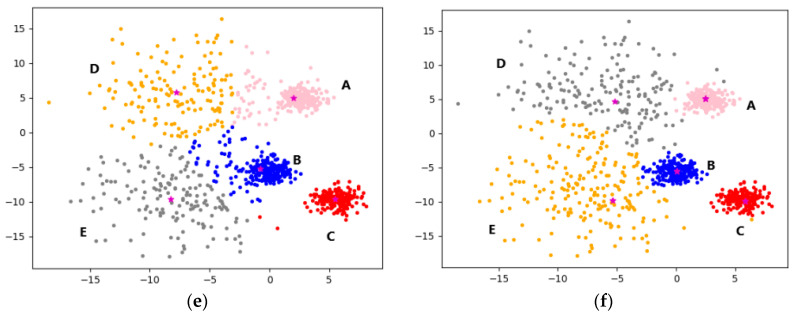
Clustering effect comparison chart. (**a**) DPC (*n* = 1000, C = 8). (**b**) DPC (*n* = 1000, C = 5). (**c**) Reference [17] (*n* = 1000, C = 5). (**d**) K-means (*n* = 1000, C = 5). (**e**) Reference [18] (*n* = 1000, C = 5). (**f**) F-DPC (*n* = 1000, C = 5).

First, the DPC is tested, and the default parameters are selected. Due to the influence of the multi-density data, the data will be automatically divided into eight categories, as shown in Figure 9a. According to the eight cluster centers, it can be seen that the clustering effect is very poor. For better comparison with other algorithms, manually set the number of DPC cluster centers equal to 5, and the clustering results are shown in Figure 9b. It can be seen from the figure that most of the data are clustered correctly, and especially the clustering result of class C is the best, because the distribution of class C is far from other classes. The boundary data of class A and class B of the original data intersect with the boundary data of class D and E, so that this kind of multi-density data cannot accurately divide the category intersection area for a global parameter. Similarly, this problem also exists in the intersection area of the upper and lower data of D and E, and the clustering effect is not stable due to the empirical setting of parameters. If the parameter setting is too large, it should be mistakenly classified into two types, otherwise, the two types will be mistakenly classified into one type.

Experiments were carried out on reference [17], the default dataset of this algorithm is 5 categories, as shown in Figure 9c. It is obvious from the figure that when there are intersections between clusters of different densities, the clustering effect is significantly improved, and most of the data categories are basically clustered correctly. However, at the intersection of the boundaries of D and E, there are still some data that cannot identify clusters of different densities due to sensitive parameters.

In experiments on the K-means algorithm, manually set the K-means parameter to 5, and continuously calculate the mean for iteration to finally reach the local optimal solution, as shown in Figure 9d. As can be seen from the figure, the classes marked B and C in the original dataset are divided into one class, and the classes C and D in the original dataset are divided into three classes; this is because the selection of the initial centroid is random, and the constant iterative update causes the centroid to shift, so the clustering result is also random, and it is difficult to achieve a good clustering effect.

Experiments were performed on reference [18], and the clustering results are shown in Figure 9e. As can be seen from the figure, the clustering effect of this method is better. The combination of DPC and K-means can better obtain the initial cluster center point, which greatly improves the local optimal clustering result, and the effect is also very stable. The disadvantage is the same as that of K-means. Although the selection of the initial cluster center is optimized, it is affected by the multi-density of samples, resulting in the deviation of the cluster center, which will also affect the clustering effect.

Experiments on the F-DPC algorithm are carried out, and the clustering results are shown in Figure 9f. It can be seen from the figure that the five categories are basically clustered correctly, and the clustering effect of the data at the boundary is also significantly better than the above algorithm. The reason is that the F-DPC algorithm firstly divides the data of different densities well, and then uses different DPC parameters for different divisions to re-cluster, which overcomes the limitation of subjectively determining the cluster center that leads to poor clustering effect.

#### 4.2.2. Experimental Analysis from the Perspective of Clustering Quality

This section uses three shapes of multi-density synthetic datasets to conduct experimental analysis from the perspective of clustering quality, and compares four clustering evaluation metrics from ARI, AMI, FMI, and NMI.

**Figure 10:**
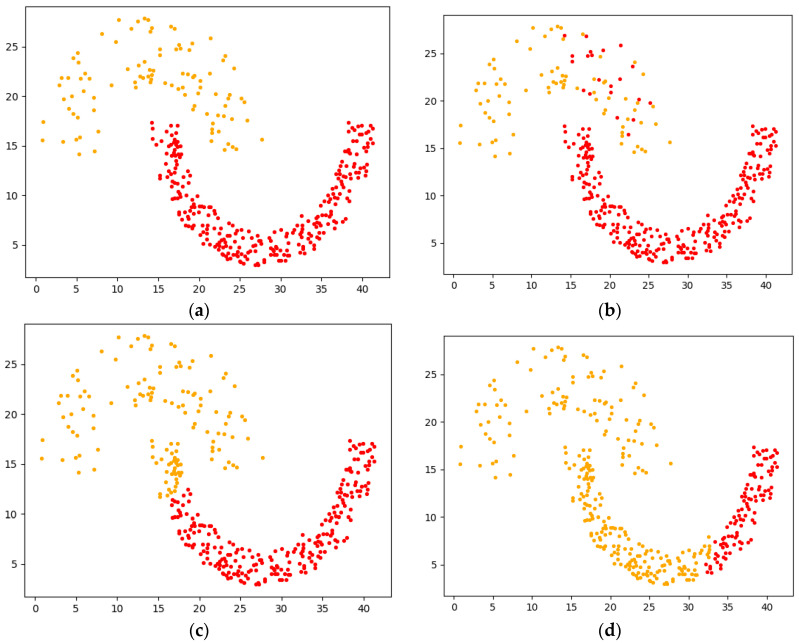

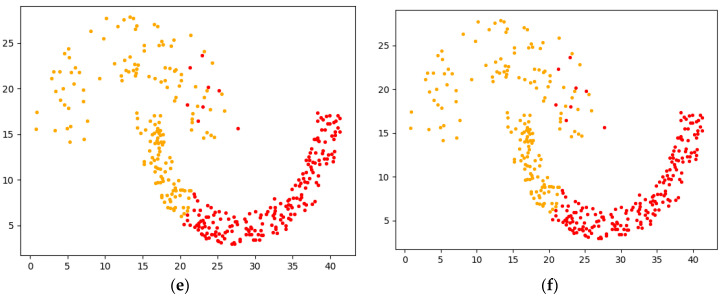
Visual analysis of Jain dataset. (**a**) Original. (**b**) F-DPC. (**c**) DPC. (**d**) Reference [17]. (**e**) Reference [18]. (**f**) K-means.

Experiment 1 uses the crescent-shaped Jain dataset for comparison, in which the Jain dataset has 373 samples and two cluster centers. The visualization results of each algorithm clustering are shown in Figure 10.

**Figure 11:**
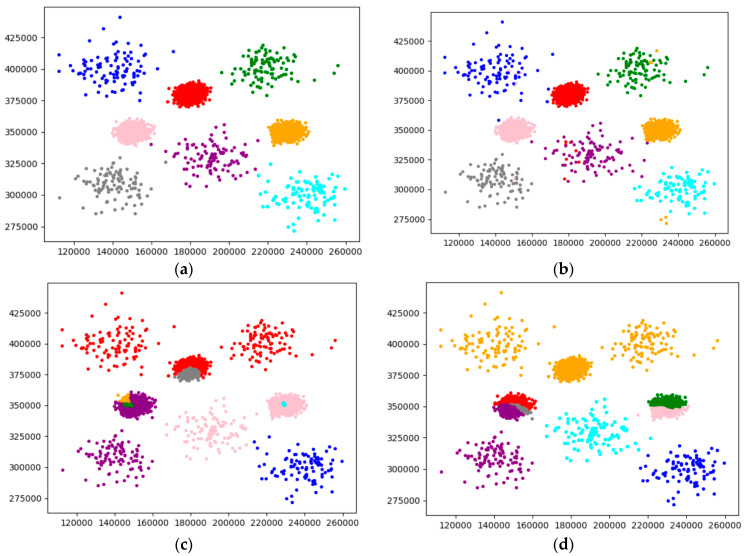

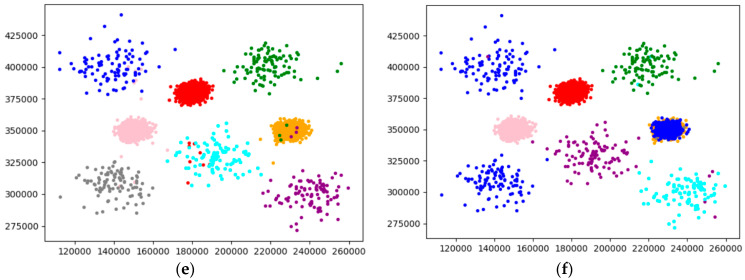
Visual analysis of Unbalance dataset. (**a**) Original. (**b**) F-DPC. (**c**) DPC. (**d**) Reference [17]. (**e**) Reference [18]. (**f**) K-means.

Experiment 2 uses the Unbalance dataset with a large number of samples for comparison, in which the Unbalance dataset has 6500 samples and eight cluster centers. The visualization results of each algorithm clustering are shown in Figure 11.

**Figure 12:**
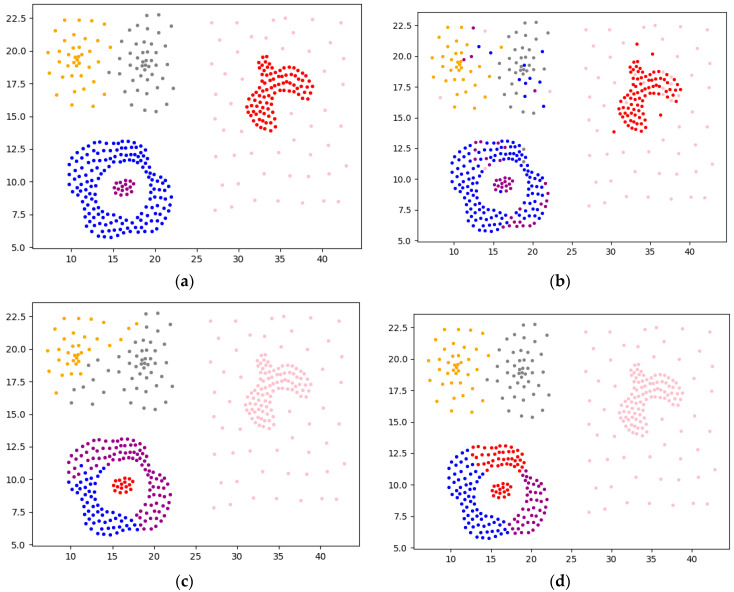

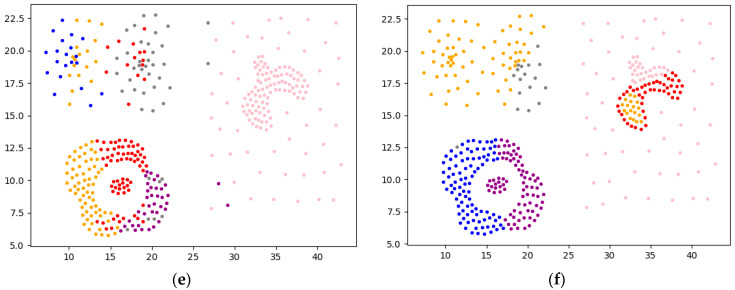
Visual analysis of Compound dataset. (**a**) Original. (**b**) F-DPC. (**c**) DPC. (**d**) Reference [17]. (**e**) Reference [18]. (**f**) K-means.

Experiment 3 uses a complex Compound dataset for comparison, where the Compound dataset has 399 samples and six cluster centers. The visualization results of each algorithm clustering are shown in Figure 12.

As can be seen from Figure 10, Figure 11 and Figure 12, the overall effect of F-DPC is relatively superior, and only a few samples are classified incorrectly. However, for DPC and reference [10], for some complex annular data, it is difficult to use a parameter to correctly cluster the samples without partitioning. The method of the initial centroid optimized by the DPC algorithm and the random selection of the initial centroid of the K-means algorithm in reference [18] will be affected by the sample centroid due to continuous iteration, reducing the clustering evaluation index. According to the above experimental calculations, the evaluation index values of various datasets are drawn and charts are drawn, as shown in Table 1.

**Figure 13:**
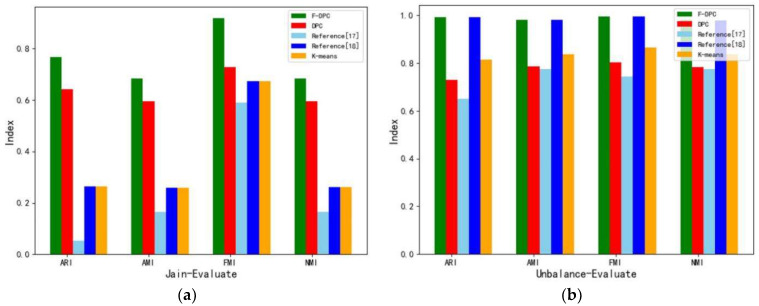

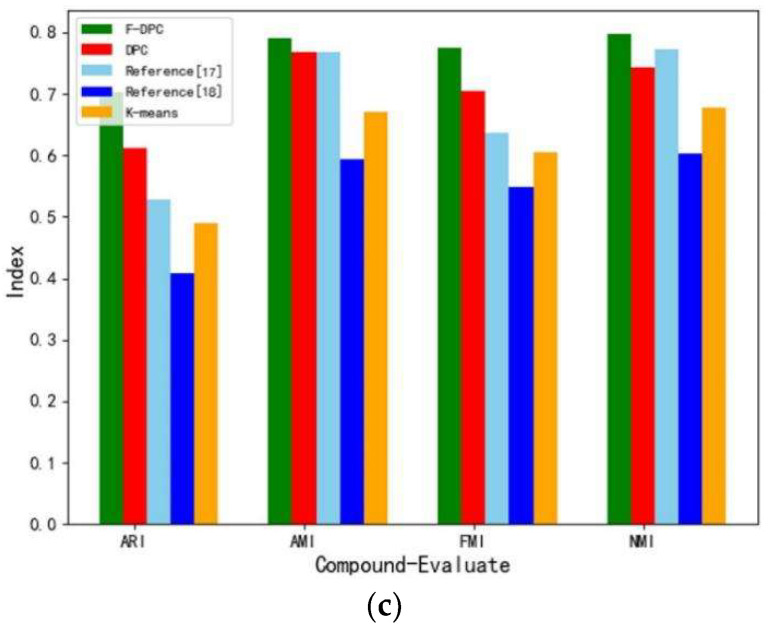
Clustering evaluation metrics for different datasets [17,18]. (**a**) Jain-Evaluate. (**b**) Unbalance-Evaluate. (**c**) Compound-Evaluate.

According to Table 1, the evaluation index bar chart is drawn to compare the clustering results of various algorithms for datasets with different shapes, as shown in Figure 13a–c. After comparison, it is found that the evaluation index of the F-DPC algorithm is the best among the three synthetic datasets with different shapes.

#### 4.2.3. Experimental Analysis from the Perspective of Sample Size

In this section, the five algorithms are tested on the multi-density datasets with 500, 1000, 2000, 5000, 10,000, and 20,000 datasets respectively, and various evaluation index values and time of the clustering results are obtained. The results are shown in Table 2.

**Figure 14:**
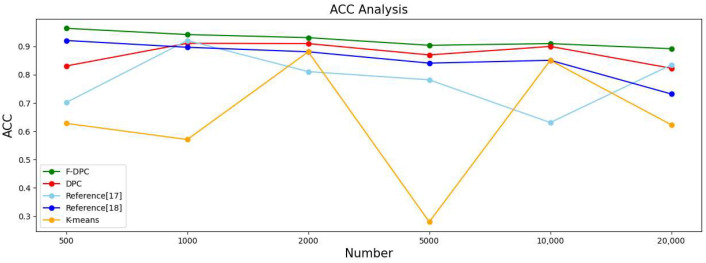
Line chart of evaluation index ACC [17,18].

**Figure 15:**
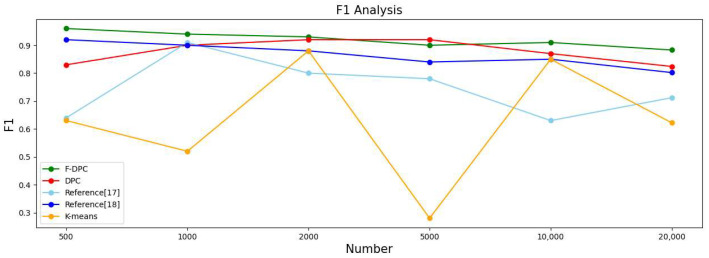
Line chart of evaluation index F1 [17,18].

**Figure 16:**
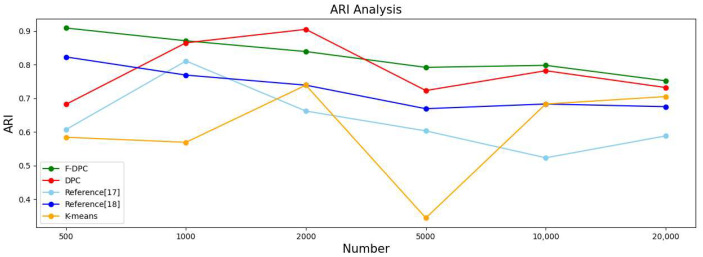
Line chart of evaluation index ARI [17,18].

**Figure 17:**
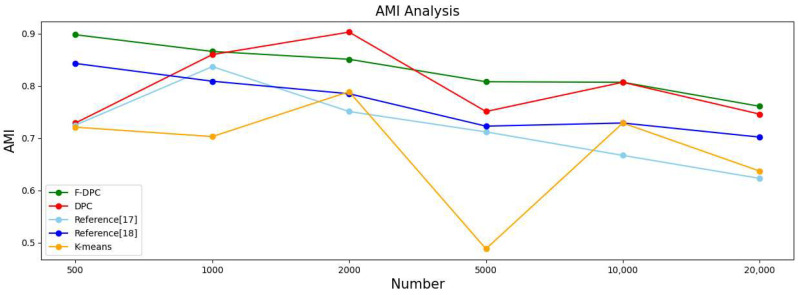
Line chart of evaluation index AMI [17,18].

In order to observe the advantages and disadvantages of the clustering quality of each algorithm more clearly, a line graph of each indicator is generated according to each indicator data in Table 2, as shown in Figure 14, Figure 15, Figure 16 and Figure 17, respectively. It can be seen from Figure 14 that the accuracy rate ACC index of the F-DPC algorithm is the highest in each data amount. It can be seen from Figure 15 that at the data volume of 5000, the F1 index of the DPC algorithm is slightly higher than the F1 index of the F-DPC algorithm, and the F1 index of the F-DPC algorithm is optimal at the remaining data volume. It can be seen from Figure 16 that at the data volume of 2000, the ARI index of the DPC algorithm is higher than that of the F-DPC algorithm, and the ARI index of the F-DPC algorithm is optimal at all remaining data volumes. It can be seen from Figure 17 that at the data volume of 2000, the AMI index of the DPC algorithm is higher than that of the F-DPC algorithm, and the AMI index of the F-DPC algorithm is optimal at all remaining data volumes.

In general, the K-means algorithm and references [17,18] have poor clustering quality, while F-DPC has the best clustering quality. The clustering quality of DPC is slightly lower than that of F-DPC. In the F1 index at the data volume of 5000, and the ARI and AMI indicators at the data volume of 2000, the DPC algorithm is slightly better than the F-DPC algorithm. The reason is that the DPC algorithm selects parameters manually by default globally, while F-DPC selects parameters by calculation. For multi-density, the manual default parameter clustering index of DPC is contingent and unstable. On the contrary, F-DPC clusters the algorithm by calculating different parameters for different densities of datasets, which greatly improves the accuracy of the algorithm. Therefore, the overall quality of the F-DPC algorithm is the best.

#### 4.2.4. Experimental Analysis from the Perspective of Time Consumption

**Figure 18:**
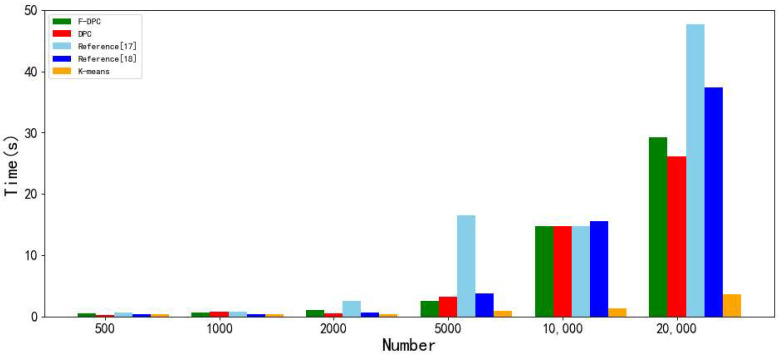
Histogram of running time of five clustering algorithms [17,18].

According to the time consumption data in Table 2, it is converted into a bar chart as shown in Figure 18.

As can be seen from Figure 18, the K-means algorithm always consumes the least time as the amount of data increases. The algorithm in [17] increases the time consumption the most with the increase in the amount of data, and it wastes time to improve the parameters of this algorithm. Reference [18] combines DPC with K-means. With the increase in the amount of data, the time consumption of the algorithm also increases rapidly. The time consumption of the DPC algorithm and the F-DPC algorithm is relatively in the middle; however, it also shows a continuous growth trend with the increase in the number of datasets, but the growth is slow.

### 4.3. Analysis of Experimental Results on UCI Real Datasets

This section selects five kinds of UCI real datasets to test and compare the clustering effect of F-DPC in real-world datasets, and finally draws a chart for the clustering result indicators of each algorithm. Table 3 shows the relevant information of the five UCI datasets selected in this experiment, which are different in terms of quantity, dimension, and number of classes. Finally, ACC, ARI, AMI, and FMI are used as algorithm evaluation indicators. The experimental results are shown in Table 4.

It can be seen from Table 4 that F-DPC performs relatively best among the five UCI datasets. In addition, the algorithm uses the K-nearest neighbor idea to divide the dataset and adaptively selects the advantages of cluster centers, which makes it significantly higher than other algorithms among many indicators.

## 5. Conclusions

Aiming at the limitation that the DPC algorithm has only one parameter globally, this paper proposes an F-DPC clustering algorithm for processing multi-density data. Firstly, according to the idea of K-nearest neighbors, the bifurcation point is found to divide the dataset, and the *d_c_* parameter corresponding to each division is recalculated, which solves the defect caused by one global parameter. The cluster center is determined by the largest discontinuity point in the divided area, and the original classical DPC relies on subjective factors to find the cluster center point. After performing DPC clustering on each division, the cluster center points of each division are optimized according to the fusion rules, and the optimized cluster centers are used for clustering. The clustering results of each division are integrated and then the clusters are fused to prevent redundant clusters generated by too many K-nearest neighbors. Experiments show that the F-DPC algorithm has the best clustering effect and the best clustering quality.

This paper proposes that when the F-DPC algorithm uses K-nearest neighbors to divide data, manually setting the bifurcation point may lead to too many divisions. However, the final algorithm can use the fusion of clusters to improve the redundantly divided dataset. If a clear calculation method can be used to determine the number of divisions, the calculation amount of the algorithm can be greatly reduced, and the accuracy of the algorithm can be improved. In the future research, for the algorithm, the divided data will use a clear calculation method for the division times of the multi-density data, reducing the division times and finally replacing the fusion technology of the algorithm, which can reduce the computational complexity of the algorithm. After the dataset is divided, the parameter calculation can be processed in blocks by using big data technology, which can also reduce the running time of the algorithm. Therefore, optimizing the number of times of dividing the dataset and adopting the distributed execution algorithm are the next research priorities. In terms of application, many scholars have used the improved density peak clustering algorithm to achieve good clustering effect and application performance in many engineering fields, such as traffic congestion prediction, vehicle distance detection, user privacy protection, personalized recommendation, etc. [53,54,55,56,57,58,59,60,61]. The next step of this algorithm will be combined with real-life application cases to solve real-life and other related problems.

## Figures and Tables

**Table 1:** Evaluation of clustering indicators for each dataset.

Algorithm	Jain				Unbalance				Compound			
	ARI	AMI	FMI	NMI	ARI	AMI	FMI	NMI	ARI	AMI	FMI	NMI
F-DPC	**0.768**	**0.684**	**0.918**	**0.685**	**0.994**	**0.982**	**0.995**	**0.981**	**0.703**	**0.791**	**0.774**	**0.797**
DPC	0.643	0.595	0.729	0.595	0.731	0.787	0.804	0.783	0.612	0.769	0.705	0.744
Reference [17]	0.052	0.164	0.591	0.166	0.650	0.774	0.743	0.775	0.527	0.767	0.637	0.772
Reference [18]	0.265	0.260	0.674	0.262	0.993	0.981	0.995	0.980	0.409	0.594	0.548	0.602
K-means	0.265	0.260	0.674	0.262	0.816	0.837	0.866	0.838	0.490	0.671	0.605	0.677

**Table 2:** Evaluation metric values and times on different numbers of datasets.

Algorithm	500					1000					2000				
	ACC	F1	ARI	AMI	Time(s)	ACC	F1	ARI	AMI	Time(s)	ACC	F1	ARI	AMI	Time(s)
F-DPC	**0.964**	**0.963**	**0.909**	**0.898**	0.52	**0.942**	**0.941**	**0.871**	**0.866**	0.73	**0.931**	**0.932**	0.839	0.851	1.12
DPC	0.831	0.831	0.682	0.729	0.34	0.911	0.902	0.865	0.860	0.79	0.910	0.910	**0.905**	**0.903**	0.53
Reference [17]	0.702	0.643	0.607	0.725	0.68	0.921	0.911	0.811	0.837	0.83	0.811	0.804	0.662	0.751	2.59
Reference [18]	0.921	0.921	0.823	0.843	0.43	0.897	0.891	0.769	0.809	0.44	0.881	0.881	0.739	0.785	0.72
K-means	0.628	0.632	0.584	0.721	0.41	0.571	0.524	0.569	0.703	0.39	0.881	0.881	0.739	0.785	0.49
**Algorithm**	**5000**					**10,000**					**20,000**				
	ACC	F1	ARI	AMI	Time(s)	ACC	F1	ARI	AMI	Time(s)	ACC	F1	ARI	AMI	Time(s)
F-DPC	**0.904**	**0.904**	**0.792**	**0.808**	2.62	**0.910**	**0.909**	**0.798**	**0.807**	14.71	**0.892**	**0.883**	**0.752**	**0.761**	29.24
DPC	0.870	0.873	0.723	0.751	3.34	0.900	0.901	0.782	0.807	14.82	0.823	0.824	0.732	0.746	26.12
Reference [17]	0.782	0.767	0.603	0.712	16.51	0.631	0.542	0.523	0.667	14.81	0.835	0.712	0.588	0.623	47.62
Reference [18]	0.841	0.830	0.669	0.723	3.86	0.851	0.836	0.683	0.729	15.61	0.732	0.802	0.675	0.702	37.33
K-means	0.280	0.211	0.344	0.488	0.96	0.851	0.836	0.683	0.729	1.32	0.623	0.622	0.705	0.637	3.62

**Table 3:** Information about the UCI dataset.

Datasets	Number	Feature	Class
Iris	150	4	3
Wine	178	13	3
Seed	210	7	3
Vowel	871	3	6
WDBC	569	30	2

**Table 4:** UCI real datasets clustering evaluation.

Algorithm	Iris				Wine				Seed			
	ACC	ARI	AMI	FMI	ACC	ARI	AMI	FMI	ACC	ARI	AMI	FMI
F-DPC	**0.898**	0.722	0.720	**0.824**	**0.552**	**0.355**	**0.378**	**0.560**	0.788	**0.962**	**0.971**	**0.985**
DPC	0.876	0.702	0.719	0.801	0.491	0.154	0.150	0.467	0.611	0.332	0.467	0.641
Reference [17]	0.891	**0.731**	**0.767**	0.822	0.501	0.143	0.176	0.438	0.623	0.443	0.578	0.667
Reference [18]	0.888	0.716	0.738	0.811	0.542	0.133	0.175	0.437	**0.982**	0.972	0.960	0.981
K-means	0.890	0.716	0.739	0.811	0.540	0.132	0.175	0.438	0.981	0.929	0.898	0.953
**Algorithm**	**Vowel**				**WDBC**							
	ACC	ARI	AMI	FMI	ACC	ARI	AMI	FMI				
F-DPC	**0.356**	**0.372**	**0.502**	**0.499**	**0.690**	**0.688**	**0.624**	**0.723**				
DPC	0.241	0.352	0.469	0.497	0.689	0.663	0.605	0.625				
Reference [17]	0.242	0.313	0.465	0.459	0.628	0.613	0.589	0.622				
Reference [18]	0.143	0.314	0.438	0.441	0.519	0.466	0.483	0.469				
K-means	0.233	0.290	0.446	0.422	0.501	0.422	0.428	0.482				

## Data Availability

The artificial dataset in this paper comes from P. Fänti and S. Sieranoja K-means properties on six clustering benchmark datasets: Applied Intelligence, 48 (12), 4743–4759, December 2018 https://doi.org/10.1007/s10489-018-1238-7 BibTex (accessed on 1 August 2022). The UCI datasets used in this article are from the UCI Machine Learning Repository https://archive.ics.uci.edu/ml/datasets.php (accessed on 6 August 2022).
